# Exploring spatial understanding and cognitive load using ultrasound in learning cardiac anatomy: A pilot study

**DOI:** 10.1002/ase.70118

**Published:** 2025-09-08

**Authors:** Johanna Maria de Lange, Karin J. Baatjes, Wouter Willaert, Janine C. Correia

**Affiliations:** ^1^ Division of Clinical Anatomy, Department of Biomedical Sciences, Faculty of Medicine and Health Sciences Stellenbosch University Cape Town South Africa; ^2^ Division of Surgery, Department of Surgical Sciences, Faculty of Medicine and Health Sciences Stellenbosch University Cape Town South Africa; ^3^ Department of Human Structure and Repair Ghent University Ghent Belgium; ^4^ Department of Gastrointestinal Surgery, Faculty of Medicine and Health Sciences Ghent University Ghent Belgium

**Keywords:** cardiac anatomy, cognitive load, medical education, spatial understanding, ultrasound

## Abstract

Although ultrasound (US) appears to complement traditional anatomy teaching, limited objective data exist on its efficacy. Existing literature often relies on student perceptions rather than performance‐based outcomes. Additionally, the role of spatial understanding (SU)—the ability to mentally manipulate and interpret 3D anatomical relationships—and cognitive load (CL)—the mental effort required to learn—remains underexplored in the context of US‐based instruction. The study consisted of three parts, with assessments before and after the US session. Prior to the session, students completed two paper‐based tests on SU and cardiovascular system (CVS) anatomy. During the session, cardiac anatomy was explored through an introduction to US physics, a practical demonstration, and hands‐on practice. Post‐session, SU and CVS knowledge were reassessed, and participants completed a CL Scale Questionnaire. Thirty‐one students participated in the study. Pre‐ and post‐testing of CVS anatomy knowledge showed a mean increase of 11.33% (*p* < 0.05), while participants' mean SU scores improved from 65.71% to 81.04% (*p* < 0.05). The highest student rating on the CL Scale was observed when measuring the germane load, specifically the item assessing perceived learning (8.55 ± 1.31), while the lowest rating was reported for measurement of extraneous load, particularly the item assessing distractions (1.23 ± 1.61). This study provided insightful reports on the efficacy of US on SU and CL in anatomy education, showing its potential to improve learning outcomes and prepare students for clinical practice.

## INTRODUCTION

Anatomy is a fundamental basic science, essential for understanding pathophysiology and clinical applications.^1^, ^2^ A solid grasp of anatomical structure–function relationships enhances physical examinations, diagnostic imaging, and surgical procedures. Ideally, anatomy curricula should integrate systematic, functional, and topographic perspectives.^3^ Traditionally, anatomy is taught through lectures and cadaver dissections,^4^, ^5^ but these methods pose challenges, particularly in visualizing complex three‐dimensional (3D) relationships, such as in cardiac anatomy. Additionally, limitations in body donation programs, cultural and religious restrictions, and resource constraints affect cadaver‐based learning.^6^, ^7^

Ultrasound (US) is a safe, portable, and non‐invasive imaging modality that offers real‐time visualization of anatomical structures, increasingly adopted in medical education to promote clinical relevance and enhance spatial learning. In contrast to modalities like CT and MRI, US enables immediate, dynamic interaction with live anatomy, making it especially suitable for bedside and procedural instruction.

Several medical schools now incorporate US into anatomy instruction as a complementary tool.^1^, ^2^, ^8^, ^9^, ^10^, ^11^, ^12^, ^13^ By offering real‐time visualization, US deepens students' grasp of anatomy and fosters an appreciation of “living anatomy”.^14^, ^15^, ^16^, ^17^, ^18^, ^19^, ^20^ However, while studies highlight students' positive perceptions and self‐reported knowledge gains from US,^1^, ^2^, ^8^, ^9^, ^10^, ^11^, ^12^, ^13^ empirical evidence on its cognitive impact remains limited. The mechanisms by which US influences learning and spatial reasoning are not yet well understood.

Spatial understanding (SU) plays a crucial role in anatomical learning.^21^, ^22^, ^23^, ^24^ It involves recognizing 3D structures, recalling their spatial relationships, and mentally manipulating them—key skills for tasks such as surgery and medical imaging.^22^, ^23^ A distinctive characteristic of anatomy is the mental manipulation of visual objects, in addition to the study of various representations and relations of those objects (e.g., radiographs and sonograms). Therefore, numerous studies have highlighted the strong connection between SU and the learning of anatomy.^19^, ^21^, ^22^, ^24^, ^25^, ^26^, ^27^, ^28^, ^29^ Since US relies on interpreting organ size, shape, and motion,^30^ strong SU may enhance students' ability to learn through US, whereas lower SU could impose additional cognitive demands.^21^, ^31^, ^32^, ^33^ Hands‐on US training may help mitigate these challenges.

Cognitive load theory (CLT) is central to this study, defining cognitive load (CL) as comprising intrinsic CL (ICL), extraneous CL (ECL), and germane CL (GCL).^34^, ^35^, ^36^, ^37^, ^38^, ^39^ ICL relates to task complexity and cannot be altered, but instructional design can manage its impact.^36^, ^38^ ECL, influenced by content delivery and instructional format, can be minimized to improve learning efficiency.^39^, ^40^ GCL refers to the effective transfer of knowledge into long‐term memory, facilitated by well‐structured learning strategies.^36^, ^37^ Given the complexity of anatomy, excessive CL can hinder learning,^35^, ^41^ underscoring the need for integrating clinical applications to enhance relevance.^41^ Spatial understanding can influence the level of CL, particularly for tasks requiring mental manipulation of structures in 3D space, making the interaction between SU and CL a critical consideration in instructional design.

Ultrasound is increasingly used in anatomy education, but its effectiveness depends on maintaining an optimal balance of ICL, ECL, and GCL.^37^ While US may improve visualization and comprehension, novice learners may experience cognitive overload, particularly those with lower SU.^33^

This study aimed to: (1) evaluate the impact of US‐based instruction on students' understanding of cardiac anatomy; (2) assess changes in students' SU after a hands‐on US session; and (3) examine the CL associated with US learning, given established associations between these variables and visuospatial ability in existing literature. This was based on previous research indicating that visuospatial ability may vary across sex and age, affecting how learners engage with 3D anatomical content.^42^, ^43^

## MATERIALS AND METHODS

The study was conducted at the Division of Clinical Anatomy, Department of Biomedical Sciences, in the Faculty of Medicine and Health Sciences, at Stellenbosch University and employed a quantitative study design. The study population included first‐, second‐, and third‐year Bachelor of Medicine and Bachelor of Surgery (MBChB) students (approximately 900 students) enrolled at Stellenbosch University for the 2023 academic year. These students were included because they all had prior knowledge of the cardiovascular system (CVS) anatomy, which was scheduled in their undergraduate medical curriculum. In addition, they also had prior US experience, as all the medical groups had been exposed to US during their CVS anatomy module. Bachelor of Medicine and Bachelor of Surgery students from any other academic year groups, as well as postgraduate students at the FMHS at Stellenbosch University, were excluded from the study.

The sampling strategy for the exploration of SU and CL using US in learning cardiac anatomy was that of voluntary response. Since not every individual in the population had the same probability of being included in the sample, voluntary response sampling is a non‐probability sampling method. The final sample comprised potential respondents who were interested and willing to participate in the proposed study.^2^

### Data collection

Ethical approval for this study was obtained by Stellenbosch University's Health Research Ethics Committee (HREC) (reference number S23/04/073, date of approval August 11, 2023), which complies with the requirements stipulated in the National Health Act (Act 61 of 2003). In addition, institutional research permission and permission from the MBChB programme committee were obtained (date of permissions May 29, 2023, and August 24, 2023, respectively).

The study was conducted in three different parts, with testing done prior to and after the hands‐on practical US session. Two paper‐and‐pencil tests were administered to the participants prior to the hands‐on practical US session, assessing SU and Cardiac Anatomy knowledge, respectively. The pre‐ and post‐Cardiac Anatomy tests consisted of 12 questions that required the participants to identify the different parts of the heart in a cross‐sectional diagram, as well as three questions which required identification of different regions of the heart on a US image. The tests were identical in format and content to ensure consistency in difficulty. A sample test is provided under Supporting Information. Inter‐ and intra‐observer agreement was ensured through independent re‐scoring of a subset of tests (10%), with 100% agreement confirmed manually.

The mental rotation test (MRT) consisted of 24 questions, with every individual question consisting of one model and four alternatives (two of which are correct and two of which are incorrect). With every question, participants were tasked with finding the counterparts (two pieces) of 3D objects that had been rotated at different angles and directions. There was only a difference in rotation between the correct and target figures. There were two distracter figures remaining that depicted mirror versions of the target figure or rotated copies of a different target figure.

These tests were provided simultaneously to all participants. They were instructed on how to proceed with each test according to recommended time limits based on validated testing protocols. Time limits were based on validated testing protocols for each assessment to ensure consistency and minimize cognitive fatigue. Incomplete tests were scored based only on the responses completed within the allotted time. When the time limit for the Cardiac Anatomy pre‐test was exceeded, the Principal Investigator (PI) collected the papers before the commencement of the MRT, which served as an assessment for SU.

During the US session, cardiac anatomy was explored, as ultrasonography allows for the dynamic evaluation of the heart.^44^ The anatomy course coordinator and the PI established and outlined the objectives of the US session in advance. Furthermore, the hands‐on practical US session did not interfere with the participants' scheduled academic classes and was scheduled in accordance with their academic timetable, which took into consideration their clinical rotations, scheduled tests, and commencement of new modules.

The student‐centered didactic framework for the US session followed the model of preparation, linking, hooking, engagement, and transfer (PLHET), as presented by Jurjus and colleagues.^45^ Table 1 illustrates the PLHET model, the components, its purpose, and what was accomplished during the US session.

**TABLE 1:** The PLHET Process.^45^

Session component	Purpose	Hands‐on practical US session
Preparation	Provided participants with background information and set expectations.	Videos, practical notes and learning objectives were shared with the volunteering participants, prior to the session, via e‐mail.
Linkage	Stimulated participants' learning: linked what was to be learned to what students already knew and/or had experienced.	Referenced to the information from their anatomy lectures and practical sessions.
Hook	Stimulated and kept the participants interested by showing the relevance of the material to their work	Clinical scenarios relevant to Cardiac Anatomy were discussed during the session. The session was guided by a Family Physician, an Emergency Physician, and a Cardiac Physiologist, henceforth known as instructors.
Engagement	Had participants apply the material, integrating it with their prior knowledge/skills, and acquiring new knowledge/skills.	The demonstration of the relevant structures was done by the instructors using the US devices.
Transfer	Enhanced the retention of new learning by having participants apply it to a previously unknown situation.	Participants used the US devices themselves and identified the relevant Cardiac Anatomy structures.

A theoretical context and expectations were set during the preparation phase.^45^ As part of the preparation component, videos, practical notes, and learning objectives were shared with the volunteering participants via e‐mail. This was done prior to the hands‐on practical US session. According to Kameda et al.,^11^ the CL of students should not be exacerbated by providing them with excessive amounts of information all at once. Providing the participants with introductory material before the US session enabled them to become more familiar with the basic principles of US, which maximized the learning potential of the US demonstration and hands‐on component.^6^ Furthermore, image acquisition proficiency is predicated upon a comprehensive understanding of sonography principles, in conjunction with adequate practice of guided US scanning.^46^ The two videos, which were provided as hyperlinks via e‐mail, were the following: “Clarius: Fundamentals of US 1 (Physics)” (https://www.youtube.com/watch?v=cI7ULKNhVcw) and “Cardiac US: Basic Windows and Anatomy” (https://www.youtube.com/watch?v=8fOUM98P2Sc). As a result of the linkage component, the participants were able to understand the information that was presented to them during the theory and practical sessions of their CVS anatomy module.

During the first 30 min of the two‐hour session, the participants were divided into three groups, and the instructors briefly introduced the respective groups to the basics of US physics and knobology. The latter was followed by US practical demonstrations about CVS anatomy. This formed part of the engagement phase. The demonstrations were conducted on volunteering participants, who provided informed consent (online) prior to the hands‐on practical US session. However, as mentioned in the literature, there was the possibility of identifying or finding existing pathology during the scanning.^11^, ^46^, ^47^, ^48^ Therefore, by providing informed consent, the volunteers acknowledged that the risk existed that abnormal US findings could be revealed in front of fellow participants. No such abnormalities were identified. However, should this have occurred, the instructors would have confidentially informed them of the finding(s) immediately after the US session had ended and arranged for an appropriate clinical follow‐up, as required.^46^, ^47^

The practical demonstrations were done with two U‐Image handheld, wireless US devices, and one cart‐based US machine (Versana Active™). It was conducted under optimal lighting conditions in a dissection hall in the Division of Clinical Anatomy, Department of Biomedical Sciences, FMHS, Stellenbosch University, with image projection on audio‐visual equipment of excellent quality. The latter enabled the participants to clearly visualize and gain an appreciation of the anatomical structures shown.^6^

The demonstration included a description of each window, a 2D view, as well as Doppler modes for CVS imaging. Different views on the US apparatuses provided information about the different anatomical structures of the heart, including the ventricles, atria, valves, and sub‐valvular mitral apparatus and how they function dynamically.^42^ Blood flow in arterial, venous, and intracardiac vessels could also be studied with color Doppler US, while transaortic and trans‐mitral blood flow patterns could be displayed using pulsed‐wave Doppler.^44^ During the demonstration, participants had the opportunity to be actively engaged, ask questions about CVS anatomy and ultrasonography. Furthermore, to keep the students interested and engaged (hook phase), various clinical scenarios were discussed to highlight the relevance of anatomy and clinical settings.

As part of the transfer phase, participants were allowed to have hands‐on practice using US devices to visualize various CVS anatomy structures. They were divided into three smaller groups to facilitate access to the US devices and to enable approximately 20–25 min of hands‐on scanning time per participant. The objective was to include, in each small group, a minimum of two participants who indicated their biological sex as male (M) and who had volunteered prior to the hands‐on practical US session to act as live models. Participants were also able to practice scanning on themselves, and during the practical US session, instructors were present to assist participants and answer any questions. Even though an instructor‐to‐participant ratio of 1:4 has been proposed in recent literature as a method to support the learning process during the hands‐on US scanning, due to volunteer availability, this study had an instructor‐to‐participant ratio of approximately 1:10. All participants attended the same US session.

Three days after the hands‐on practical US session, two paper‐and‐pencil tests were once again administered to the participants, assessing SU and Cardiac Anatomy knowledge, respectively. A three‐day interval between the learning session and post‐tests was introduced to assess knowledge retention beyond immediate recall. The tests were completed in one of the BMRI's lecture halls at the FMHS. The content of and time limit for both tests (30 min) were the same as the tests administered prior to the hands‐on practical US session. In addition, the participants completed a CL Scale (CLS) Questionnaire,^34^ consisting of 10 items: three measuring ICL (items 1–3), three ECL (items 4–6), and four GCL (items 7–10), using a 10‐point Likert scale.

### Data analysis

The PI performed all statistical analysis under the guidance and assistance of a trained biostatistician from Stellenbosch University's Biostatistics unit, as well as a statistician affiliated with Stellenbosch University. Analysis of all statistical data were computed by using computer software, namely R‐Studio (2024.07.0 Build 548), IBM SPSS® (Version 29.0.0.0), and Microsoft® Excel® (Version 2312 Build 17126.20132).

Descriptive statistics (percentage, frequency, mean, standard deviation (SD), median, interquartile range (IQR), skewness, kurtosis, and standard error (SE)) were used to analyze quantitative data from both the Cardiac Anatomy Tests, the MRTs, as well as the CLS Questionnaire. Thereafter, the normality of the data was determined by conducting a Shapiro–Wilk normality test, as the number of participants were less than 50. Where the data distribution was normal, differences between pre‐ and post‐test scores were determined by using paired‐ and student *t*‐tests. Where the data were not normally distributed, differences were determined by Mann–Whitney, Chi‐squared, and Wilcoxon signed‐rank tests. Relationships between dependent and independent variables were determined by performing a multivariate analysis of variance (MANOVA), while correlations were explored using a correlation heatmap. For all analyses, a *p*‐value < 0.05 was considered statistically significant for paired *t*‐tests.

Inter‐observer agreement was ensured through independent re‐scoring of a subset of tests by a Senior Lecturer in the Division of Clinical Anatomy, Department of Biomedical Sciences, FMHS, Stellenbosch University, who did not form part of the research team, while the PI ensured intra‐observer agreement. Inter‐ and intra‐observer agreement of the pre‐ and post‐Cardiac Anatomy and SU tests, as well as the CLS Questionnaire, was assessed on 10% of the dataset. A minimum of two weeks was ensured before intra‐observer agreement was assessed on the dataset. Moreover, marking methods used were shown and explained to the inter‐observer prior to assessment.

## RESULTS

### Demographic information

73.81% of the 42 participants who completed the pre‐tests continued to the hands‐on US session and post‐tests. Among them, 22 (70.97%) chose their biological sex as female, and nine (29.03%) as male, with none opting to withhold their biological sex. Participants had a median age of 19 years (IQR: 19–20; range: 18–23). The group included 21 (67.74%) MBChB I students (13 females, 8 males), 8 (25.81%) MBChB II students (7 females, 1 male), and 2 (6.45%) MBChB III students (both female). 31 of the 900 eligible students (3.44%) participated in the full study.

### Cardiac anatomy test

**FIGURE 1:**
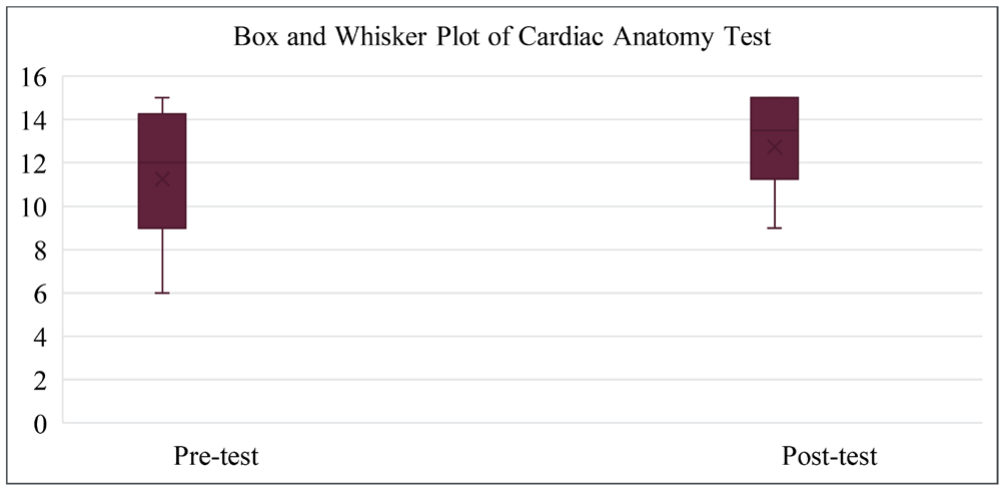
Box and Whisker plot of cardiac anatomy test.

The mean score was 11.65 ± SD 2.27 (77.67%) for the pre‐test and 13.35 ± SD 1.52 (89%) for the post‐test (range: 6–15 and 9–15, respectively). The pre‐ and post‐test scores' quartiles are shown in Figure 1.

Furthermore, the mean of correct answers for the pre‐test CVS cross section was 10.58 ± SD 1.77 (88.17%) and 11.39 ± SD 0.88 (94.92%) for the post‐test. The mean of correct answers for the CVS sonograph was 1.06 ± SD 1.21 (35.33%) for the pre‐test and 1.97 ± SD 1.35 (65.67%) for the post‐test.

Given the small sample size, a Shapiro–Wilk test was used to assess data normality, revealing a significant deviation (*W* = 0.93, *p* = 0.04). Median scores between pre‐ and post‐tests showed a significant difference (*W* = 260.5, *p* = 0.02). A non‐parametric Kruskal–Wallis test comparing academic year groups also showed significant difference (Chi‐squared = 6.62, *p* = 0.04).

### Spatial understanding

#### Overview of the results

**FIGURE 2:**
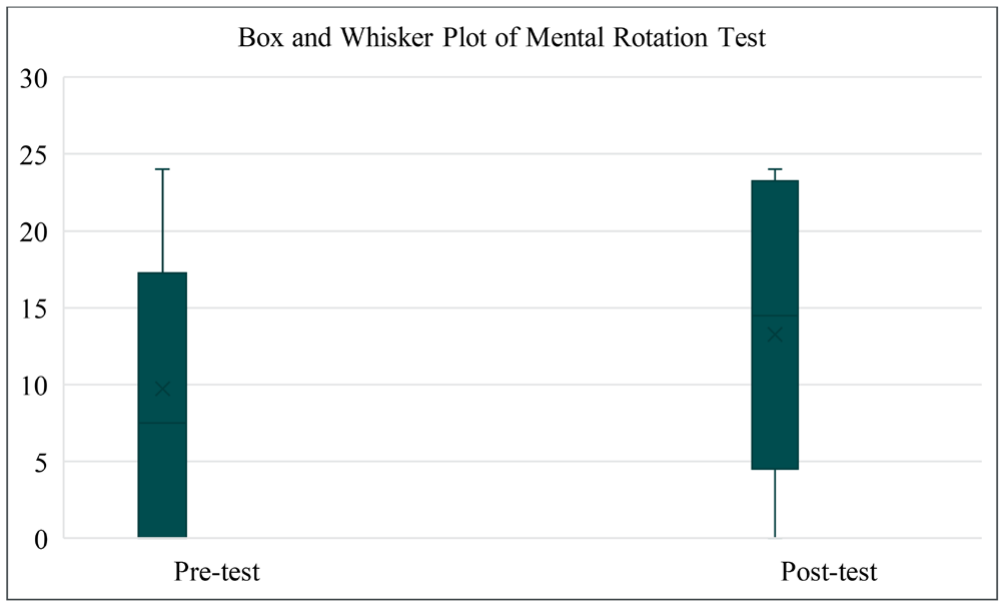
Box and Whisker plot of Mental Rotation Test.

The mean of correct answers for the pre‐MRT was 15.77 ± SD 5.52 (65.71%) and 19.45 ± SD 5.38 (81.04%) for the post‐MRT, with the lowest and largest number of correct responses being 0 to 24 and 6 to 24, respectively. The initial and post‐MRT scores' quartiles are shown in Figure 2. Overall, 38.71% (12/31) of the pre‐test scores were 75% or greater, while 77.42% (24/31) of the post‐test scores fell in that category.

The analysis of the pre‐ and post‐MRT scores, by means of a Shapiro–Wilk test, did not show evidence of non‐normality (*W* = 0.96; *p* = 0.22). Thus, a parametric test was conducted, and the results showed a statistically significant difference between the pre‐ and post‐MRT scores (*W* = 254; *p* = 0.001).

#### Spatial understanding and age

The exploration of the relationship between SU and age using US in teaching and learning cardiac anatomy was one of the secondary objectives of this study. The different academic year groups were used as an indication of age in the statistical analysis. There were no significant correlations between age and MRT scores (Kruskal–Wallis chi‐squared = 0.19; *F* = 0.12; *p* = 0.91).

#### Spatial understanding and biological sex

The mean pre‐MRT scores for males and females were 18.44 (SD 5.73) and 14.68 (SD 5.18), respectively. When comparing mean MRT scores for the post‐testing phase, the results showed mean scores of 21.44 (SD 4.48) and 18.64 (SD 5.59) for males and females, respectively. When combining the results, they showed no statistically significant difference between males and females regarding their pre‐ and post‐MRT scores (*p* = 0.39). Table 2 displays a summary of the results.

**TABLE 2:** Summary of the results of the pre‐ and post‐mental rotation scores (biological sex).

	Pre‐MRT		Post‐MRT		Difference between MRT scores	
	Male	Female	Male	Female	Male	Female
Mean	18.44	14.68	21.44	18.64	3.00	3.95
Median	21.00	15.50	23.00	20.50	2.00	5.00
SD	5.73	5.18	4.48	5.59	2.60	2.97
Skewness	−0.97	−1.42	−1.77	−1.20	0.23	−0.35
Kurtosis	−0.31	1.37	1.80	0.08	−1.70	−0.88
SE	1.91	1.10	1.49	1.19	0.87	0.63

### Cognitive load scale questionnaire

In Table 3, we present the means of the CLS items as well as their SDs, medians, skewness, kurtosis, and SEs. The ICL score (mean 4.34, SD 2.49) was less than average, indicating that the participants perceived a low level of content complexity. The ECL score (mean 1.37, SD 1.34) was also below average, indicating minimal exposure to distracting elements during the US session. The self‐perceived learning (GCL) score (mean 8.16, SD 1.38) was relatively high on the 10‐point scale, indicating strong self‐perceived learning following the US session.

**TABLE 3:** Summary of the Cognitive Load Scale Questionnaire results.

Items	Mean (SD)	Median	Skewness	Kurtosis	SE
Item 1	3.68 (2.52)	3.00	0.14	−1.32	0.45
Item 2	4.19 (3.05)	4.00	0.01	−1.24	0.55
Item 3	5.16 (3.05)	5.00	−0.22	−0.94	0.55
Item 4	1.48 (1.81)	1.00	1.23	0.87	0.32
Item 5	1.23 (1.61)	1.00	1.28	0.44	0.29
Item 6	1.39 (2.20)	1.00	2.26	4.55	0.40
Item 7	8.55 (1.31)	9.00	−0.87	0.16	0.24
Item 8	7.81 (1.83)	8.00	−0.57	−0.45	0.33
Item 9	7.77 (1.87)	8.00	−0.30	−1.23	0.34
Item 10	8.52 (1.43)	9.00	−0.65	−0.65	0.26
ICL	4.34 (2.49)	4.67	−0.31	−1.03	0.45
ECL	1.37 (1.34)	1.00	0.76	−0.39	0.24
GL	8.16 (1.38)	8.25	−0.25	−1.20	0.25

Moreover, when reporting on the asymmetry of the data distribution, the majority of the data points have a negative skew, which extends to more negative values. All but one of the data points (Item 6) has a kurtosis of less than three. Thus, the distribution is platykurtic (tends to produce fewer and less extreme outliers than the normal distribution). All the data points have a small SE, which indicates the closeness of the means. It is thus more likely that the mean of the sample is an accurate representation of the true population mean.

The analysis of the CLS Questionnaire, specifically pertaining to the ICL, did not show evidence of non‐normality (*W* = 0.94; *p* = 0.11). However, the analysis of the ECL and GCL showed that the distribution departed significantly from normality (*W* = 0.88; *p* = 0.002 and *W* = 0.93; *p* = 0.04, respectively). When comparing the CLs of males (ICL – 4.89 ± 2.10; ECL – 1.41 ± 1.27; GCL – 7.81 ± 1.66) and females (ICL – 4.12 ± 2.64; ECL – 1.35 ± 1.40; GCL – 8.31 ± 1.27), similar results were obtained. The latter was also seen when analyzing the CLs of the three different academic year groups, namely MBChB I (ICL – 4.48 ± 2.24; ECL – 1.78 ± 1.36; GCL – 7.58 ± 1.26), MBChB II (ICL – 4.08 ± 3.35; ECL – 0.21 ± 0.31; GCL – 9.38 ± 0.76), and MBChB III (ICL – 4.00 ± 2.36; ECL – 1.67 ± 1.41; GCL – 9.38 ± 0.18).

### Spatial understanding and cognitive load

The analysis of SU and CL showed that the distribution departed significantly from normality (*W* = 0.830; *p* < 0.001 and *W* = 0.924; *p* = 0.031, respectively). The relationship between SU and CL was explored through Spearman's rank correlation. Based on this analysis, SU and CL showed a non‐significant negative correlation (*r* = −0.227; *p* = 0.220) with a small effect size. Furthermore, the findings revealed a moderate positive correlation (*r* = 0.47, *p* < 0.05) between GCL and post‐session SU scores, suggesting that students who experienced higher germane processing also demonstrated greater improvements in SU. Conversely, ECL showed a weak negative correlation (*r* = −0.22, *p* = 0.08) with SU scores, highlighting the detrimental impact of extraneous demands on spatial learning outcomes. These results confirm that instructional designs aiming to reduce ECL and enhance GCL can significantly bolster SU in anatomy education.

## DISCUSSION

### Aim one

The primary aim of this study was to explore the effect of using US on undergraduate medical students' cardiac anatomy knowledge. For example, one question required the participants to identify the left atrium in the given cross‐sectional image of the heart. Pre‐ and post‐test assessments demonstrated significant improvement, aligning with prior research that highlights US's effectiveness in enhancing anatomical comprehension.^11^, ^14^, ^15^, ^16^, ^17^, ^18^, ^49^ Unlike static images or cadaver dissections, US provided real‐time visualization, reinforcing students' understanding of cardiovascular structures.^14^

Cardiac anatomy poses unique challenges for US learning due to its rhythmic motion and complexity of flow‐based imaging. Unlike MSK anatomy, which often involves static structures, cardiac anatomy demands interpretation of dynamic 2D and Doppler‐based views, potentially increasing cognitive and psychomotor demands.

Authentic learning theory supports these findings, emphasizing the importance of real‐world applications in medical education.^50^ Ultrasound technology fosters active learning, shifting students from passive observers to engaged participants. By integrating new knowledge with existing cognitive schemas, students consolidate information into a more retrievable form.

Knowledge transfer from the US session likely improved post‐test scores. Transfer occurs when learning in one context enhances performance in another.^51^, ^52^, ^53^ The similarity between the US session (cross‐sectional visualization) and the post‐test (cross‐sectional 2D and US images) likely facilitated this process. By contrast, Vandenbossche et al.^53^ found that a significant disparity between learning and assessment hindered student performance in MSK anatomy. However, their findings may not be generalizable to our study due to contextual differences.

A key goal of anatomy education is to facilitate knowledge transfer to clinical practice.^51^ Ultrasound enhances SU, a critical skill for interpreting imaging, performing procedures, and surgical navigation.^30^, ^54^ Studies have shown that US‐trained students exhibit better diagnostic accuracy and procedural skills.^25^, ^26^ However, its benefits in clinical settings vary. If not implemented correctly, US may increase CL, limiting its effectiveness.^55^ Hashemiparast et al.^55^ found that students sometimes struggled to apply US‐acquired knowledge under clinical pressure and doubted their abilities. Thus, a balanced approach is necessary to maximize learning without overwhelming students. Furthermore, US may not be equally effective in all contexts. Technical complexity, limited instructor availability, and institutional barriers (e.g., access to devices) may reduce its feasibility or efficacy.

Well‐designed assessments can help achieve this balance. Integrating US‐based evaluations, 3D models, and spatial reasoning exercises can enhance learning without unnecessary cognitive burden. Structured Objective Clinical Examinations (OSCEs) incorporating US‐guided tasks may further reinforce practical application. Additionally, peer and self‐assessment could promote reflection and self‐directed learning.

A longitudinal assessment approach, tracking students' progress over time, would support continuous anatomical competence rather than isolated achievements. This strategy encourages deeper learning, fostering both conceptual understanding and clinical application of anatomy.

### Aim two

This study's second aim was to examine the impact of US on undergraduate medical students' SU in learning cardiac anatomy. To achieve this, SU was assessed before and after exposure to US. Results revealed variability in SU abilities among participants prior to the hands‐on session, aligning with previous findings that conventional methods (e.g., lectures, textbooks) often fail to support 3D anatomical visualization.^14^, ^15^, ^16^, ^56^ The complexity of anatomy necessitates more interactive learning approaches.

A significant improvement in SU was observed post‐session, demonstrating US's potential as a valuable adjunct in anatomy education. The hands‐on, real‐time nature of US enhances students' ability to visualize spatial relationships, a skill essential for sonographic proficiency.^21^, ^31^, ^32^ These findings align with other studies reporting US's benefits in improving anatomical SU.^14^, ^19^, ^21^, ^22^, ^25^, ^26^, ^57^ For example, Kourdioukova et al.^25^ and Griksaitis et al.^14^ found US superior to traditional methods in fostering spatial comprehension, likely due to its dynamic and real‐time depiction of anatomical structures.^4^, ^19^, ^56^

Weimer et al.^21^ demonstrated that US training improved students' visual–spatial abilities, anatomical knowledge, and radiological interpretation skills. This study's improvements may stem from several US‐specific advantages: real‐time image manipulation facilitating deeper SU, active engagement reinforcing knowledge retention, and the integration of tactile and visual stimuli enhancing learning efficiency.

While MRT scores increased, visual–spatial abilities remain an important factor in anatomy performance.^27^ Although practice effects have been reported,^58^, ^59^ prior research suggests minimal gains with repeated MRT exposure,^60^ making it unlikely that practice alone influenced results.

Our findings contrast with some studies,^20^, ^53^ where US‐based teaching showed slightly lower performance than traditional methods, though differences were not statistically significant.^20^, ^53^ These discrepancies highlight the importance of instructional design and adequate guidance in US‐based learning. Close supervision in this study may have contributed to the better outcomes, underscoring the need for structured implementation.

Cognitive load considerations are crucial in conjunction with SU. While US enhances SU through intuitive visualization, poorly implemented sessions could increase ECL.^61^ The structured, supervised session in this study likely mitigated ECL, allowing participants to focus on spatial relationships rather than technological challenges. De Lange et al.^12^ emphasized the importance of well‐organized US training, as disorganized sessions hinder learning. The low mean ECL score (1.23 ± 1.61) in this study suggests minimized distractions, optimizing working memory for SU tasks.

The hands‐on US session facilitated dynamic engagement with live anatomical images, reinforcing spatial information in long‐term memory and bridging theoretical knowledge with clinical applications. The interplay between CL and SU underscores the benefits of real‐time visualization and cognitive strategies for effective learning.

This study also explored SU differences based on biological sex. While males scored higher in both pre‐ and post‐tests, the difference was not statistically significant, consistent with prior research.^44^, ^46^, ^53^, ^62^, ^63^ Some studies report significant male advantages in SU,^44^, ^46^, ^53^, ^62^, ^63^ often attributed to greater exposure to spatial learning activities.^43^ However, SU is trainable,^27^ and this study suggests US‐based training may help students with lower initial SU improve their proficiency.

### Aim three

The third aim of this study was to assess the effect of US on CL in teaching cardiac anatomy. We evaluated whether US imposed greater cognitive demands, thereby facilitating or hindering learning. Our findings indicate a nuanced impact on CL.

Learning CVS anatomy with US did not significantly increase ICL. The heart's complexity remains a challenge regardless of the instructional method. However, US provided real‐time, 3D visualization, allowing some participants to regulate ICL more effectively by engaging prior knowledge and reducing content volume, consistent with Hickam et al.^39^ A few participants, however, reported increased ICL, likely due to the difficulty of interpreting US images and integrating them with existing anatomical knowledge—aligning with Talip et al.,^41^ who noted high ICL in anatomy due to its interconnected content. Some participants also struggled with US's novelty alongside extensive theoretical material.^41^, ^53^, ^61^

Ultrasound reduced ECL by replacing static 2D textbook images, which require mental reconstruction into 3D. US's dynamic visualization streamlined this process, enabling students to focus on anatomical relationships rather than struggling with abstraction. Similar findings by Dreher et al.^26^ showed that US integration reduced ECL while enhancing student satisfaction and performance.

Participants reported high GCL, indicating deep cognitive processing. Ultrasound's hands‐on nature and immediate visual feedback facilitated stronger mental models of cardiac structures, enhancing comprehension. Improved cardiac anatomy test and MRT scores further suggest increased GCL, aligning with CLT's emphasis on mental effort in learning.^39^

Our results showed a moderate positive correlation between GCL and SU, and a weak negative correlation between ECL and SU. These findings suggest that minimizing distractions (ECL) and encouraging meaningful engagement (GCL) may particularly benefit learners with lower baseline SU.

The psychomotor domain is integral to US training. Mastery of knobology, probe handling, and spatial navigation of live images require not only cognitive but physical skills, reinforcing the importance of repeated hands‐on exposure. Image acquisition requires manual dexterity, spatial coordination, and fine motor skills. Engaging with the US device may reinforce anatomical relationships through embodied learning, similar to how dissection supports retention via physical interaction. Future work should more explicitly assess the role of psychomotor engagement in enhancing spatial reasoning and anatomical knowledge retention.

According to CLT, new tools like US can elevate CL both intrinsically and extraneously.^59^ However, our results indicate that US's pedagogical benefits outweigh potential CL increases. Ultrasound optimizes cognitive resources by enhancing SU, supporting Sweller et al.'s^64^ emphasis on managing CL for optimal learning.

Our findings contrast with Jamniczky et al.,^61^ where students experienced cognitive strain due to US's complexity, particularly knobology. In our study, pre‐session videos, notes, and learning objectives introduced US principles, minimizing information overload and reducing CL.^11^ This approach aligns with Jamniczky et al.,^65^ who found lower CL when students received prior US training.

The CLS Questionnaire results highlight the potential for integrating US into anatomy curricula to improve learning efficiency. Beyond visual engagement, US fosters cognitive engagement, reinforcing its value as a teaching tool.

## LIMITATIONS

This study was conducted at a single institution (Stellenbosch University) within a specific curriculum, limiting the generalizability of findings. Different organizational and didactical settings may yield different results, and future studies should consider diverse cultural environments, larger urban settings, and alternative remote teaching platforms.

The small sample size did not fully represent MBChB I–III cohorts, limiting the strength of conclusions. However, the biological sex distribution (70% female, 30% male) reflects that of these cohorts. Additionally, self‐selection bias may have influenced results, as motivated volunteers do not necessarily represent the broader student population.

The study lacked a control group, making it difficult to attribute observed changes solely to the educational intervention. Causation cannot be determined, and intervention effectiveness may be overestimated. Moreover, 11 of the original 42 students (26.19%) dropped out, and test–retest effects may have contributed to score improvements, as participants could have learned from the pre‐test itself.

Although Doppler mode was briefly demonstrated to illustrate dynamic blood flow, it was not a focus of instruction or assessment. Doppler may introduce unnecessary complexity for novice learners, and the research team recommends that future studies limit its use in introductory sessions.

Prior exposure to US in the CVS anatomy module may have influenced CL, as the practical session was not their first US experience. The session covered select CVS anatomy topics but did not reflect a full anatomy course, limiting extrapolation to other regions. The cardiac anatomy test included both gross anatomy images and sonograms, making it difficult to isolate US's specific impact on anatomy learning.

Spatial understanding was assessed solely with the MRT, a reliable but theoretically limited measure with a ceiling effect, potentially obscuring pre‐test–post‐test differences. Additional spatial tests (e.g., Purdue Spatial Visualization Test) could have provided a more comprehensive evaluation.

The CL instrument assessed only one practical US session, rated as high quality. However, participants lacked a lower‐quality comparison, and familiarity with the Family Physician, also a Senior Lecturer, may have biased evaluations. Applying the instrument across multiple sessions would yield further insights.

Administering the CLS Questionnaire three days after the session may have captured residual rather than immediate CL. While this allowed for assessment load retention, it may not fully reflect the in‐session cognitive demands. Future studies should administer the CLS Questionnaire immediately post‐session or at multiple time points for a more accurate assessment.

Lastly, while introductory materials were provided to mitigate CL, it is unclear whether participants engaged with them. Thus, conclusions regarding CL's role in learning image interpretation remain tentative, as students struggling with anatomy may also lack the necessary knowledge to interpret US images. Additionally, administering the CLS three days post‐session may have assessed residual perceptions of load rather than immediate cognitive demands. Ideally, future studies should capture CL during or immediately after the learning activity.

## CONCLUSION

This study explored the effectiveness of US in anatomy education, focusing on SU and CL. While previous studies have investigated US's impact on either SU or CL, this study is among the first to concurrently examine both variables in a cardiac anatomy context using a multi‐measure design. This adds valuable insight into how these dimensions interact in shaping learning outcomes. Findings suggested that hands‐on US sessions may have improved students' cardiac anatomy knowledge and SU, as indicated by higher test scores and enhanced spatial reasoning. The interactive and dynamic nature of US allowed real‐time visualization of anatomical structures, which seemed to foster deeper comprehension compared with static images or cadavers.

Ultrasound also positively impacted CL by reducing intrinsic and extraneous loads while promoting germane load. Clear, real‐time visuals simplified learning, enabling participants to focus on understanding complex anatomical relationships and developing robust mental models of cardiac structures. Overall, this research underscores US as an effective educational tool that bridges theoretical and practical knowledge, enriches spatial reasoning, and fosters an engaging learning environment.

Integrating CL and SU concepts more explicitly in anatomy education can inform future curriculum development. Practical US sessions should be designed to minimize ECL through clear instructional goals, streamlined content delivery, and optimized learning environments. Furthermore, GCL should be maximized by incorporating active learning strategies, such as real‐time US practice and guided exploration of 3D anatomical relationships. Moreover, instruction should account for individual differences in SU, providing additional support for students with lower visuospatial abilities through targeted mental rotation exercises. Educators might also consider integrating structured mental rotation training or pre‐session modeling to help level the playing field.

In conclusion, the deliberate alignment of CL management strategies with SU‐focused learning outcomes not only enhances students' comprehension of anatomy but also prepares them for the spatially demanding tasks of clinical practice. This integration underscores the pedagogical value of US as a transformative tool in medical education. Future studies should compare US to traditional methods using randomized controlled designs, explore virtual US platforms, and evaluate how students transfer this knowledge into clinical performance.

## AUTHOR CONTRIBUTIONS

**Johanna Maria de Lange:** Conceptualization; investigation; methodology; software; writing – original draft; funding acquisition; visualization; writing – review and editing; project administration; formal analysis; data curation; resources. **Karin J. Baatjes:** Conceptualization; writing – review and editing; supervision; validation. **Wouter Willaert:** Conceptualization; validation; writing – review and editing; supervision. **Janine C. Correia:** Conceptualization; investigation; methodology; validation; visualization; writing – review and editing; project administration; resources; supervision; data curation.

## CONFLICT OF INTEREST STATEMENT

The authors declare no conflicts of interest.

## Supporting information


**Data S1:** Supporting Information.

## Data Availability

Data supporting the findings of the research are not publicly available due to participant confidentiality but can be obtained from the corresponding author upon reasonable request.
