# Supplementary material for: Exploring spatial understanding and cognitive load using ultrasound in learning cardiac anatomy: A pilot study
Source: Anat Sci Educ. 2025 Sep 8;18(11):1250–61. doi: 10.1002/ase.70118 (PMC12592905; doi:10.1002/ase.70118)
Supplement: Supplementary file 1 — Data S1: Supporting Information. [file ASE-18-1250-s001.pdf]

Novel Cardiac Anatomy Test

Record ID ______________________

Dear student,
Please complete the following test by providing labels for the numbers (1-15) on the cross-sectional diagram of the heart (1-12), as well as on the sonogram (13-15). You have 15 minutes to complete the test.

Student number ______________________

Biological sex
◯ Male
◯ Female

Age ______________________

Academic year group
◯ Second year MBChB
◯ Third year MBChB

Provide labels for numbers 1 - 12:

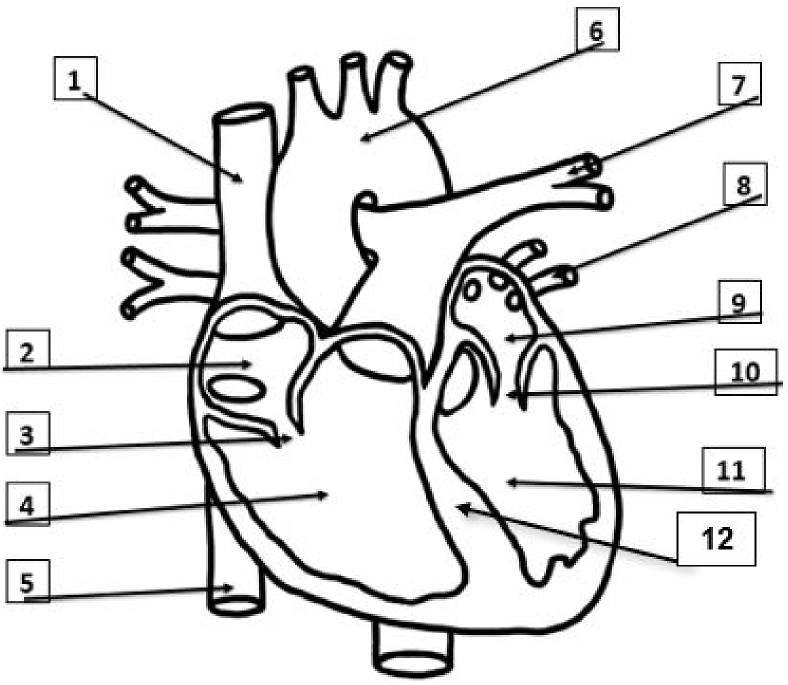

1: ______________________

2: ______________________

3: ______________________

4: ______________________________

5: ______________________________

6: ______________________________

7: ______________________________

8: ______________________________

9: ______________________________

10: ______________________________

11: ______________________________

12: ______________________________

Provide labels for numbers 13 - 15:

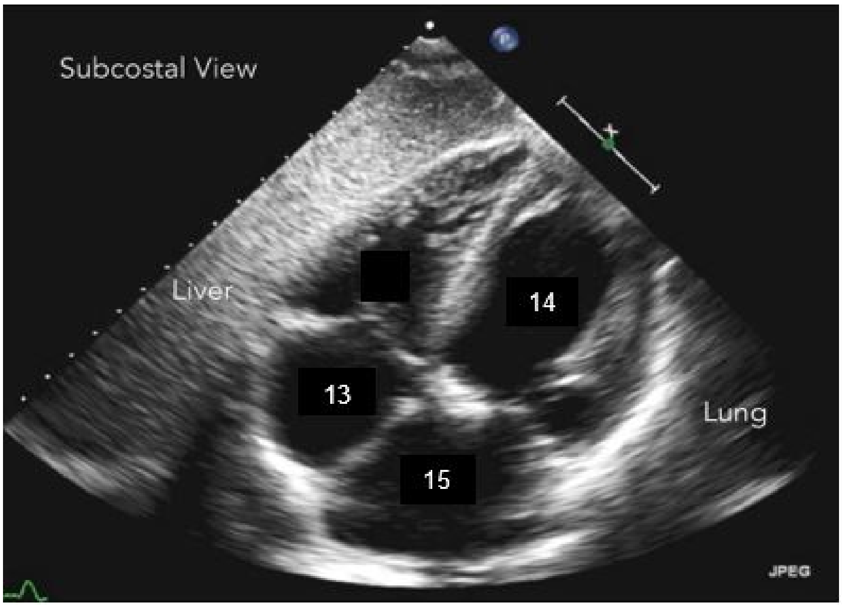

13: ______________________________

14: ______________________________

15: ______________________________
